# 4D flow cardiovascular magnetic resonance derived energetics in the Fontan circulation correlate with exercise capacity and CMR-derived liver fibrosis/congestion

**DOI:** 10.1186/s12968-022-00854-4

**Published:** 2022-03-28

**Authors:** Friso M. Rijnberg, Jos J. M. Westenberg, Hans C. van Assen, Joe F. Juffermans, Lucia J. M. Kroft, Pieter J. van den Boogaard, Covadonga Terol Espinosa de Los Monteros, Evangeline G. Warmerdam, Tim Leiner, Heynric B. Grotenhuis, Monique R. M. Jongbloed, Mark G. Hazekamp, Arno A. W. Roest, Hildo J. Lamb

**Affiliations:** 1 Department of Cardiothoracic Surgery, Leiden University Medical Center, Albinusdreef 2, 2333ZA Leiden, The Netherlands; 2 Department of Radiology, Leiden University Medical Center, Leiden, The Netherlands; 3 Department of Pediatric Cardiology, Leiden University Medical Center, Leiden, the Netherlands; 4 Department of Cardiology and Anatomy and Embryology, Leiden University Medical Center, Leiden, The Netherlands; 5 Department of Pediatric Cardiology, Utrecht Medical Center, Utrecht, The Netherlands; 6 Department of Radiology, Utrecht Medical Center, Utrecht, The Netherlands

**Keywords:** Fontan, 4D flow MRI, Viscous energy loss, FALD, Fibrosis, Exercise capacity

## Abstract

**Aim:**

This study explores the relationship between in vivo 4D flow cardiovascular magnetic resonance (CMR) derived blood flow energetics in the total cavopulmonary connection (TCPC), exercise capacity and CMR-derived liver fibrosis/congestion.

**Background:**

The Fontan circulation, in which both caval veins are directly connected with the pulmonary arteries (i.e. the TCPC) is the palliative approach for single ventricle patients. Blood flow efficiency in the TCPC has been associated with exercise capacity and liver fibrosis using computational fluid dynamic modelling. 4D flow CMR allows for assessment of in vivo blood flow energetics, including kinetic energy (KE) and viscous energy loss rate (EL).

**Methods:**

Fontan patients were prospectively evaluated between 2018 and 2021 using a comprehensive cardiovascular and liver CMR protocol, including 4D flow imaging of the TCPC. Peak oxygen consumption (VO_2_) was determined using cardiopulmonary exercise testing (CPET). Iron-corrected whole liver T1 (cT1) mapping was performed as a marker of liver fibrosis/congestion. KE and EL in the TCPC were computed from 4D flow CMR and normalized for inflow. Furthermore, blood flow energetics were compared between standardized segments of the TCPC.

**Results:**

Sixty-two Fontan patients were included (53% male, 17.3 ± 5.1 years). Maximal effort CPET was obtained in 50 patients (peak VO_2_ 27.1 ± 6.2 ml/kg/min, 56 ± 12% of predicted). Both KE and EL in the entire TCPC (n = 28) were significantly correlated with cT1 (r = 0.50, p = 0.006 and r = 0.39, p = 0.04, respectively), peak VO_2_ (r = − 0.61, p = 0.003 and r = − 0.54, p = 0.009, respectively) and % predicted peak VO_2_ (r = − 0.44, p = 0.04 and r = − 0.46, p = 0.03, respectively). Segmental analysis indicated that the most adverse flow energetics were found in the Fontan tunnel and left pulmonary artery.

**Conclusions:**

Adverse 4D flow CMR derived KE and EL in the TCPC correlate with decreased exercise capacity and increased levels of liver fibrosis/congestion. 4D flow CMR is promising as a non-invasive screening tool for identification of patients with adverse TCPC flow efficiency.

**Supplementary Information:**

The online version contains supplementary material available at 10.1186/s12968-022-00854-4.

## Introduction

The Fontan procedure provides a unique surgical solution for congenital heart disease patients with a functionally univentricular heart defect, by connecting the systemic venous return directly with the pulmonary arteries (i.e. the total cavopulmonary connection: TCPC). Consequently, the Fontan circulation requires increased central venous pressure (CVP) to overcome the serial resistance in the TCPC and pulmonary vascular bed to maintain an adequate cardiac output. The chronic exposure to elevated venous pressures provides a significant burden for upstream organs, including an almost universal occurrence of liver fibrosis [1]. Furthermore, the ability to increase preload and thereby cardiac output during exercise is limited leading to a diminished exercise capacity [2].

Being one of the few modifiable factors in the Fontan circulation, blood flow efficiency in the TCPC has been widely studied using predominantly in silico computational fluid dynamic (CFD) models [3–5]. Highly variable blood flow efficiency in the TCPC has been reported, related to the presence of energy-consuming geometric factors that can become apparent during follow-up [3, 5]. Recently, correlations were found between CFD-derived TCPC blood flow efficiency and clinical outcome, including liver fibrosis [6] and exercise capacity. [7]

Drawbacks of CFD modelling are that it is time-consuming and requires expert knowledge and infrastructure, limiting widespread use in clinical care. 4D flow cardiovascular magnetic resonance imaging (CMR) is emerging as a non-invasive screening tool for visualization of energy-consuming flow patterns in the TCPC and quantification of flow-related hemodynamic parameters including kinetic energy (KE) and viscous energy loss rate (EL) [8–10].The hypothesis in this study is that these in vivo 4D flow CMR-derived energetics in the TCPC are associated with exercise capacity and liver fibrosis/congestion. Subsequently, the aim of this study is to determine KE and EL in the TCPC using 4D flow CMR and to assess the relationship with exercise capacity and CMR-based assessment of liver fibrosis/congestion.

## Materials and methods

### Study population

Sixty-two Fontan patients were prospectively evaluated using a comprehensive cardiovascular and liver CMR research protocol, including 4D flow imaging of the TCPC, between 2018–2021 at the Leiden University Medical Center, Leiden, the Netherlands. The study was approved by the medical ethical review board of the Leiden University Medical Center (P18.024). Written informed consent was obtained from all patients and/or their parents. All patients > 8 years old without contraindications for CMR with a clinical indication for routine surveillance of the Fontan pathway using CMR were eligible for inclusion.

### Cardiopulmonary exercise testing

Cardiopulmonary exercise testing (CPET) was performed on an upright bicycle ergometer (General Electric Healthcare, Chicago, Illinois, USA) including peak oxygen uptake (VO_2_) assessment. CPET was performed on the same day or within 6 months of CMR. A continuous incremental bicycle protocol was executed according to the Godfrey protocol [11]. Peak VO_2_ (ml/kg/min) and percentage of predicted peak VO_2_ (%) were determined in all patients that achieved maximal effort (respiratory exchange ratio > 1.0) using previously reported reference values. [12].

### Laboratory data

Standard laboratory assessment was performed on the same day or within 6 months of CMR, including liver enzymes, total protein, albumin, total bilirubin, and international normalized ratio (INR). Additionally, a feces sample was tested on alfa-1-antitrypsin excretion as a marker of (early) protein losing enteropathy. Results were interpreted using the hospital’s standard age- and gender-specific reference values.

### Cardiovascular magnetic resonance

All CMR examinations were performed on a 3 T system (Ingenia, Philips Healthcare, Best, the Netherlands).

#### Multiparametric liver imaging

Patients underwent multiparametric liver imaging using proton density fat-fraction (PDFF), T2*, and iron-corrected T1 mapping (cT1) for the assessment of liver fat, iron, and fibrosis/inflammation/congestion, respectively (Liver MultiScan™, Perspectum Diagnostics Ltd., Oxford, UK) [13]. Patients underwent liver imaging after fasting for at least 4 h. No intravenous contrast agent was used. Values were calculated from segmentation of whole liver regions (cT1 and PDFF) or one or more regions of interest (T2*) on one or more transversal slices. cT1 mapping allows for quantification of extracellular fluid in the liver, which is increased in both liver fibrosis, inflammation and venous congestion. Since inflammation is usually absent in liver fibrosis in Fontan patients [14] and liver enzymes are usually normal or only mildly elevated, elevated cT1 values in Fontan patients will predominantly reflect fibrosis and/or venous congestion. Reference values of PDFF, T2* and cT1 are < 5.6%, > 12.5 ms and 633–794 ms, respectively. [15–17] These metrics have been proven to be repeatable and reproducible across different manufacturers and field strengths [18]. All liver scans were anonymized and subsequently analyzed (Perspectum, Oxford, UK) using an online portal.

#### Ventricular function

Ventricular ejection fraction and cardiac output were calculated by ventricular volume analysis (MASS software, Leiden, the Netherlands) of multislice two-dimensional cine transversal images using a balanced steady-state-free-precession sequence. The cardiac index was derived by normalizing cardiac output for body surface area (Haycock).

#### 4D flow CMR

Patients underwent retrospective electrocardiogram (ECG) and respiratory navigator-gated 4D flow CMR examination dedicated for imaging of the TCPC. Acquisition details for 4D flow CMR are covered in Additional file 1: Table S1. A velocity encoding of 80 cm/s resulted in minimal aliasing in only a few patients which could be corrected by build-in anti-aliasing software (CAAS, MR Solutions, PIE Medical Imaging BV, Maastgricht, the Netherlands). No intravenous contrast was used for the 4D flow acquisition.

A 3D reconstruction of the TCPC was semi-automatically segmented on magnitude-weighted speed images of a time-averaged reconstruction of the 4D flow velocity field (CAAS v5.1, MR Solutions, Pie Medical Imaging). The region of interest covered the area between the Fontan tunnel (above entry of the hepatic veins), superior vena cava (SVC) (below the brachiocephalic vein) and the right- and left pulmonary arteries (PAs) up to the levels of the segmental branches. The right upper lobe branch was excluded from the segmentation since the resolution of the 4D flow CMR for this small vessel is insufficient [19].

Both the KE and EL of blood flow in the TCPC were computed from the 4D flow CMR velocity field using in-house developed software, as previously described [20]. KE represents the amount of energy in the blood flow due to its motion. EL represents the rate of kinetic energy lost in the blood flow due to friction and can be computed from three-dimensional velocity gradients derived from 4D flow CMR. EL was computed using the viscous dissipation function from the Navier–Stokes equation, assuming laminar blood flow [20]. The total amount of KE (in milliJoule, [mJ]) and EL (in milliwatts, mW) within the TCPC is computed by summing voxel-wise energetics for each time-phase (24 phases per cardiac cycle). The cardiac-cycle averaged values are reported. Previous studies showed good to strong reproducibility of 4D flow based segmentation, flow quantification and quantification of hemodynamic energetics [21–24].

In patients in whom the entire TCPC was available for analysis, energetics were normalized for inflow (SVC + Fontan tunnel flow, in L/min); KE_norm_flow_ in mJ per L/min and EL_norm_flow_ in mW per L/min [25].

#### Segmental analysis

To further explore and compare the energetics in the specific components of the TCPC, a sub analysis was performed to determine segment-specific energetics (Fontan tunnel, central Fontan confluence, SVC, left PA (LPA) and right PA (RPA), using in-house developed software as previously described [25]. Additional benefit of this approach is that in patients with a fenestration closure device or PA stent in situ, in whom the entire TCPC cannot be segmented because of device-related flow artefacts, energetics in the TCPC segments without device-related artefacts can still be studied. In these patients, only part of the Fontan tunnel (distal to the device) or PA (proximal to the stent) can be included in the segmentation. Segments with a length < 1.5 cm were excluded from the analysis to ensure sufficient voxels for energetic analysis. [25].

In summary, the TCPC was automatically divided into 5 segments. Energetics within each segment were normalized for segment-specific inflow (Fontan confluence) or inflow + length (Fontan tunnel, SVC, LPA, RPA; KE_norm_flow+length_ and EL_norm_flow+length_) [25]. The additional normalization for length in these segments is required as these segments are not always completely available for analysis (e.g. due to stent or device related artefacts) making direct comparison between complete and incomplete segments not possible.

Cross-sectional areas (CSA) normalized for body surface area (BSA) of the included conduit, SVC, LPA and RPA segments were determined perpendicularly to their centerlines at a 1 mm interval [21]. The mean CSA of each segment is reported.

A flow chart of the study methods and performed analyses are reported in Additional file 1: Fig. S1.

### Statistical analysis

Continuous data are presented as mean (standard deviation) or median (interquartile range, IQR), as appropriate. Normal distributions of continuous data were tested using the Shapiro–Wilk test. Pearson and/or Spearman correlation analysis (weak 0.3–0.5, moderate 0.5–0.7, strong ≥ 0.7–0.9 and excellent > 0.9) was performed between the main endpoints (cT1 liver mapping and maximal exercise capacity) and CMR parameters; ventricular function and 4D flow energetics in the entire TCPC. Segment-specific energetics (Fontan tunnel, SVC, LPA and RPA) were compared with each other using the Kruskal Wallis test (adjusted by the Bonferroni method for multiple tests). Correlation analysis was performed between segment-specific energetics and normalized CSA. A p-value < 0.05 was considered statistically significant. Data were analyzed with SPSS (version 25.0, Statistical Package for the Social Sciences, International Business Machines, Inc., Armonk, New York, USA) and GraphPad Prism (version 8.0, GraphPad Software, La Jolla, California, USA).

## Results

### Study population

Patient characteristics are shown in Table 1. Fifty-three percent of patients were male with a mean age at CMR of 17.3 ± 5.1 years and mean time between Fontan completion and CMR of 13.6 ± 4.8 years. The majority of patients (94%) underwent Fontan completion using a (fenestrated) 16–20 mm extracardiac Goretex conduit. Fenestrations closed spontaneously or were routinely closed after Fontan completion using a fenestration closure device. One patient still had a patent fenestration at time of CMR. All but two patients were in good clinical condition (New York Heart Association class I-II).

**Table 1:** Patient characteristics

Male/female	33/29
Primary diagnosis, n (%)	
TA	14 (23)
HLHS	12 (19)
DILV + TGA	10 (16)
DORV	6 (10)
uAVSD	5 (8)
ccTGA	5 (8)
PA + IVS	5 (8)
Other	5 (8)
Dominant ventricle	
Left, n (%)	35 (56)
Right, n (%)	21 (34)
Biventricular/indeterminate, n (%)	6 (10)
Characteristics at Fontan procedure	
Previous bidrectional Glenn shunt, n (%)	
62 (100)	
Age at Fontan, years	3.7 (1.8)
Fontan technique LT/ECC	4/58
Implanted conduit size (16/18/20 mm), n	30/22/6
Fenestration, n (%)	38 (61)
Characteristics at time of CMR	
Age at CMR, years	17.3 (5.1)
Height, cm	167 (12)
BSA, m^2^	1.6 (0.3)
Time between Fontan and CMR, years	13.6 (4.8)
NYHA-class I-II, n (%)	60 (97)
Male/female, n	33/29

Values are reported as mean (standard deviation) unless otherwise specified. BSA, body surface area; TA, tricuspid atresia; HLHS, hypoplastic left heart syndrome; DILV, double inlet left ventricle; (cc)TGA, congenital corrected transposition of the great arteries; DORV, double outlet right ventricle; uAVSD, unbalanced atrioventricular septal defect; PA + IVS, pulmonary atresia with intact ventricular septum; LT, lateral tunnel; ECC, extracardiac conduit, NYHA, New York Heart Association

### CPET and laboratory

CPET results are presented in Table 2. CPET was performed in 57 patients (92%), with 50 patients achieving maximal effort. Mean peak VO_2_ was 27.1 ± 6.2 ml/kg/min, 57% ± 12% of predicted peak VO_2_.

**Table 2:** CPET results

SBP_basal,_ mmHg	124 (15)
SBP_peak,_ mmHg	172 (26)
RER_peak_	1.1 (0.09)
Power, watt	131 (37)
% predicted	68 (15)
HRrest, bpm	82 (15)
HRpeak, bpm	172 (17)
% predicted	93 (10)
HRreserve, bpm	91 (25)
Maximal exercise (n = 50)	
Peak VO2, ml/kg/min	27.1 (6.2)
% predicted	57 (12)

Values are reported as mean (standard deviation). HRpeak, maximal heart rate at peak exercise; HRreserve, maximal heart rate-resting heart rate; HRrest, resting heart rate; RERpeak, respiratory exchange ratio at peak exercise; SBPbasal/peak, systolic blood pressure at rest/peak; VO2peak, oxygen uptake at peak exercise

Laboratory results are provided in Table 3. Total protein and albumin were normal in all but one patient with refractory protein losing enteropathy. Alanine aminotransferase, Aspartate aminotransferase and gamma glutamyltransferase levels were normal or only mildly elevated in the majority of patients. Alfa-1-antitrypsin excretion in the feces was normal (n = 48) or only mildy elevated (n = 3) in all patients.

**Table 3:** Laboratory results

	n	Mean (SD)	Range	Abnormal level, n (%)
Total protein, g/L	56	71 (7)	35–83	1 (2)
Albumin, g/L	59	48 (5)	18–54	1 (2)
Aspartate aminotransferase, U/L	57	32 (10)	18–74	22 (39)
Alanine aminotransferase, U/L	58	36 (16)	15–111	14 (24)
Gamma glutamyltransferase, U/L	56	58 (40)	18–185	25 (45)
Total bilirubin, µmol/L	55	15 (10)	5–49	13 (24)
INR*, (reference ≤ 1.2)	42	1.1 (0.1)	1.0–1.5	3 (7)
Fecal alfa-1-antitrypsine, mg/g (reference < 0.4)	51	0.2 (0.1)	0.1–0.7	3 (6)
NT-proBNP	57	113 (99)	23–446	7 (12)

^*^Patients on oral anticoagulation were excluded. SD, standard deviation. INR, international normalized ratio; NT-proBNP, N-terminal pro brain natriuretic peptide

### Comprehensive CMR analysis

#### Multiparametric liver imaging

Multiparametric liver imaging was performed in all patients. cT1 analysis could not be performed in one patient due to insufficient data quality. cT1 (reference 633–794 ms) was elevated in all patients (mean cT1 964 ± 63 ms, range 824–1073 ms). T2* was within normal ranges (reference value > 12.5 ms) in all patients (mean T2* 22.6 ± 3.4 ms). Mean PDFF (reference value < 5.6%) was 1.7 ± 3.3%. Only one patient had an elevated PDFF of 27.1% indicating liver steatosis.

#### Ventricular function and 4D flow CMR energetics

Mean cardiac index and ejection fraction were 3.4 ± 0.1 L/min/m^2^ and 48 ± 7%, respectively. 4D flow CMR of the TCPC was acquired in 61 patients. One patient wanted to stop with the CMR examination before acquisition of 4D flow CMR was completed. Five patients were excluded from 4D flow analysis due to insufficient image quality (excessive patient movement related, n = 4) or because of the presence of multiple central device-related artefacts affecting the majority of the TCPC. The total TCPC without device-related CMR artefacts was available for 4D flow energetic analysis in 28/56 patients (50%).

**Fig. 1:**
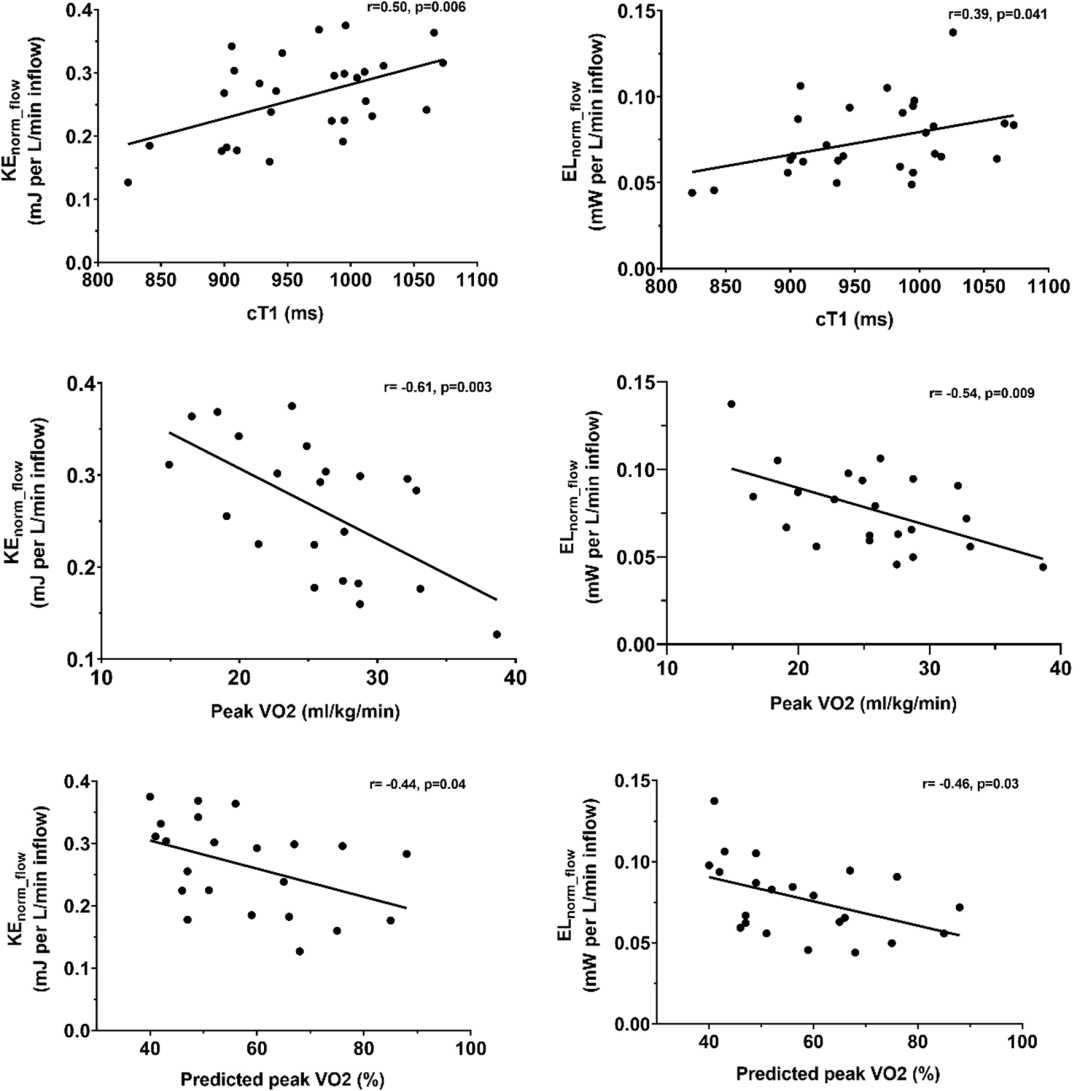
Correlation analysis between kinetic energy (KE) (left) and viscous energy loss rate (EL) (right) in the total cavopulmonary connection (TCPC) with iron corrected T1 mapping (cT1); cT1 (upper panel), peak oxygen uptake (VO_2_) (middle panel) and % predicted peak VO_2_ (lower panel) are shown

**Fig. 2:**
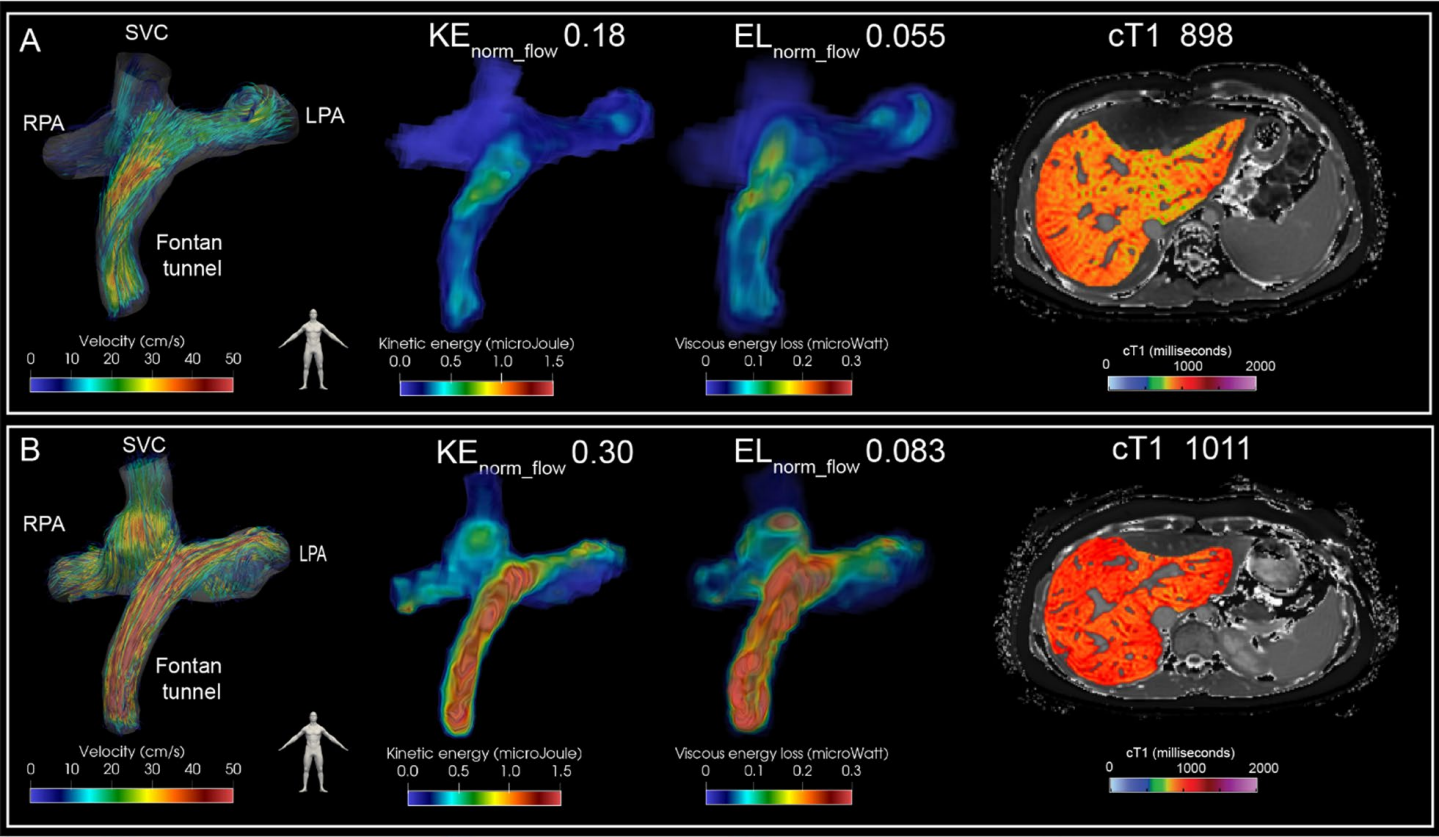
Streamline representation of blood flow in the TCPC is shown for two representative female extracardiac Fontan patients with a 16 mm Goretex conduit (left panel) for the first phase of the cardiac cycle. Corresponding spatial distribution of KE and EL is shown (middle panels). The time-averaged normalized energetics values are indicated above. Whole liver cT1 mapping is shown for a transversal slice (right panel). Note how a strong difference in blood flow velocity is present at the level of the extracardiac conduit which is strongly correlated to the areas of increased KE and EL. Patient A: 17 years old, double inlet left ventricle + transposition of the great arteries, BSA 1.5. Patient B: 18 years old, tricuspid atresia, BSA 1.8). BSA, body surface area; KE, kinetic energy; EL, viscous energy loss rate; cT1, iron-corrected T1 mapping; RPA/LPA, right/left pulmonary artery; SVC, superior vena cava

In the patients in whom the entire TCPC was available for analysis, KE_norm_flow_ and EL_norm_flow_ in the total TCPC were 0.26 ± 0.07 mJ per L/min and 0.075 ± 0.022 mW per L/min inflow. Energetics were not significantly different between genders (p = 0.27–0.37). A significant positive correlation was observed for KE_norm_flow_ and EL_norm_flow_ with cT1 (r = 0.50, p = 0.006 and r = 0.39, p = 0.04, respectively, Fig. 1). An example of TCPC blood flow patterns, related energetics and cT1 liver mapping is shown for two extracardiac Fontan patients in Fig. 2. Furthermore, KE_norm_flow_ and EL_norm_flow_ showed a significant negative correlation with peak VO_2_ (r = -0.61, p = 0.003 and r = -0.54, p = 0.009, respectively) and % predicted peak VO_2_ (r = -0.44, p = 0.04 and r = -0.46, p = 0.03, respectively, Fig. 1). No significant correlations were found between cardiac index or ejection fraction with cT1 or (% predicted) peak VO_2_. Also no significant correlations were found between ejection fraction and KE_norm_flow_ and EL_norm_flow_ (r = -0.030, p = 0.83 and r = 0.048, p = 0.725, respectively) and between cardiac index and KE_norm_flow_ and EL_norm_flow_ (r = -0.189, p = 0.16 and r = -0.147, p = 0.28 respectively).

#### Segmental 4D flow CMR energetics

Results of segmental TCPC energetics are reported in Table 4. KE_norm_flow+length_ was lowest in the SVC and lower compared to the Fontan tunnel (p < 0.001), RPA (p = 0.002) and LPA (p < 0.001). KE_norm_flow+length_ was higher in the LPA compared to the RPA (p = 0.027). EL_norm_flow+length_ was higher in the conduit (p = 0.024) and LPA (p < 0.001) compared to the SVC and higher in the LPA compared to the RPA (p < 0.001). In the Fontan tunnel, mean CSA showed a strong negative correlation with KE_norm_flow+length_ (ρ = -0.80, p < 0.001) and EL_norm_flow+length_ (ρ = -0.78, p < 0.001, Additional file 1: Table S2). A weak correlation was found between mean LPA CSA and EL_norm_flow+length_ (ρ = -0.31, p = 0.034). Energetics in the RPA and SVC were not related to segment-specific CSA.

**Table 4:** 4D flow CMR derived energetics in the TCPC

4D flow CMR	n	KE_norm_flow_	EL_norm_flow_
Total TCPC	28	0.260 (0.068)	0.075 (0.022)

*Segmental analysis*		KE_norm_flow+length_	EL_norm_flow+length_
Fontan tunnel	35	0.034 (0.011)	0.0087 (0.0029)
SVC	31	0.023 (0.007)	0.0065 (0.0024)
LPA	46	0.040 (0.018)	0.012 (0.005)
RPA	49	0.031 (0.01)	0.0081 (0.0032)
Fontan confluence	56	0.094* (0.041)	0.031* (0.013)

Values are reported as mean (standard deviation). KE_norm_flow_ and EL_norm_flow._ KE_norm_flow_ in mJ per L/min and EL_norm_flow_ in mW per L/min. KE_norm_flow+length_ in mJ per L/min per cm segment, EL_norm_flow+length_ in mW per L/min per cm segment. LPA/RPA; left/right pulmonary artery, SVC, superior vena cava; TCPC, total cavopulmonary connection; KE, kinetic energy; EL, viscous energy loss rate. * Energetic values for the Fontan confluence are normalized for flow only and thereby not directly comparable with energetics in the other four segments

## Discussion

This study for the first time shows the relationship between in vivo 4D flow CMR-derived TCPC blood flow energetics, exercise capacity and CMR-based assessment of liver fibrosis/congestion (cT1 mapping). Liver cT1 values were elevated in all patients at a mean age of 17 years, indicating universal liver fibrosis/congestion. KE_norm_flow_ and EL_norm_flow_ in the TCPC correlated with maximal exercise capacity and liver cT1, while conventional parameters as ventricular ejection fraction and cardiac index did not. The Fontan tunnel and LPA were the segments with most adverse energetics. EL_norm_flow_ was highest in patients with smallest Fontan tunnels raising concern on the current approach of Fontan completion with 16 to 20 mm rigid conduits.

Surgical construction of an energy efficient TCPC with low resistance is important to keep the increase in CVP and reduction of preload towards the single ventricle to a minimum [26]. An elevated CVP and reduced cardiac output play an important role in the occurrence of liver fibrosis and decreased exercise capacity, respectively. In silico CFD modelling studies have identified PA stenosis and undersized Fontan tunnels to be associated with reduced flow efficiency [4, 5, 27], which correlates with liver fibrosis stage and exercise capacity [6, 7]. Therefore, routine screening of Fontan patients on the occurrence of (subclinical) adverse TCPC flow efficiency is clinically relevant for timely identification of patients with adverse TCPC hemodynamics, in whom intervention may be beneficial.

Recently, 4D flow CMR has emerged as a promising non-invasive technique for quantification of in vivo TCPC flow efficiency. KE and EL in the TCPC are novel 4D flow CMR markers reflecting TCPC flow efficiency, influenced by adverse TCPC geometry (e.g. PA stenosis or undersized conduit) and related flow patterns. 4D flow CMR previously revealed adverse vortical/helical flow patterns in the Fontan confluence, PAs, or in a blind-ending pulmonary trunk, all areas associated with increased KE and EL [9, 25]. In this study, adverse 4D flow derived KE and EL in the TCPC correlated with reduced maximal exercise capacity and increased levels of liver fibrosis/congestion (cT1), and may therefore be important novel markers that can indicate adverse outcome.

### Exercise capacity

Decreased exercise capacity in Fontan patients is predominantly caused by a limited capability to maintain preload and stroke volume of the single ventricle during exercise [2]. Importantly, resting cardiac index and ejection fraction did not correlate with exercise capacity, but adverse 4D flow energetics in the TCPC did show a significant correlation. This might be explained by the fact that increased EL in the TCPC may particularly affect preload and thus cardiac output during exercise conditions only, since energy loss in the TCPC increases non-linearly with exercise [28], and are therefore not captured by evaluation of resting cardiac function parameters.

#### Liver fibrosis

Presence of Fontan associated liver disease, including liver fibrosis, is universal in Fontan patients and the chronic exposure of the liver to an elevated CVP plays an important role [1]. The association between energetics and liver cT1 mapping can therefore be explained by the fact that elevated EL in the TCPC will require an increased CVP to maintain cardiac output. Fibrosis progression rate varies between patients and factors that lead to accelerated liver fibrosis are unknown as serial liver fibrosis assessment is lacking [29]. Future longitudinal studies with liver cT1 mapping could verify if patients with adverse TCPC energetics show faster progression in liver fibrosis.

#### Liver cT1 mapping

Gold standard liver biopsy is subject to sampling errors and its invasiveness prohibits routine serial assessment. In this study a novel non-invasive approach was used based on iron-corrected T1 mapping to quantify increased levels of extracellular liver fluid, which is elevated in liver fibrosis and/or venous congestion. Although cT1 values showed good correlation with histologic liver fibrosis in adult patients with liver disease [15], validation in Fontan patients is currently lacking. Especially venous congestion will influence T1 values in Fontan patients since increased CVP has been correlated with increased levels of liver T1 [30]. Therefore, distinction between liver fibrosis or congestion due to increased CVP on elevated cT1 cannot be determined. However, since an increased CVP is associated with important morbidity such as PLE and liver fibrosis, measurement of cT1 as a combined marker of fibrosis/CVP is a promising non-invasive marker for adverse outcome.

#### Segmental analysis

Analysis of the TCPC segments that most severely affected the adverse energetics of the entire TCPC indicated that the Fontan tunnel and LPA are the areas to focus on for improved TCPC efficiency. Increased KE and EL in the conduit, carrying 70% of total systemic venous return, can be explained by the presence of undersized extracardiac conduits (IVC-conduit velocity mismatch) caused by somatic overgrowth [8], since a strong inverse correlation between conduit CSA and energetics was shown. LPA hypoplasia/stenosis, often observed in hypoplastic left heart syndrome patients, but also the presence of vortical flow in larger LPAs or in patients in whom the pulmonary trunk has not been detached, can explain the adverse energetics in the LPA. [9, 25].

## Limitations

Compared to CFD, 4D flow CMR underestimates “true” energy loss and only captures relatively large-scale flow structures due to a limited spatial resolution. However, the relative performance of the TCPC between patients remains intact making comparison of TCPC energetics between Fontan patients still possible [31]. 4D flow CMR also does not capture the influence of respiration on TCPC blood flow [32], which could significantly affect energy losses [33]. Novel 5D flow CMR sequences can be of interest to study the effect of respiration on TCPC energetics [34]. TCPC energetics could only be linked to current cT1 values in this study. Since the occurrence of liver fibrosis is presumably time-related, correlation of TCPC energetics with longitudinal cT1 mapping will be of interest and is subject to future research. Liver cT1 mapping has been shown to predict mortality and liver-related events in patients with chronic liver disease [35], but its prognostic value in Fontan patients is currently unknown. Finally, energetics in the Fontan confluence segment could not be directly compared with the other four segments since Fontan confluence energetics were normalized for inflow only. But factors that influence Fontan confluence efficiency can be still be determined, and previously significantly elevated EL in the Fontan confluence was observed when vortical flow is present.

## Conclusion

Adverse 4D flow CMR derived kinetic energy and viscous energy loss rate in the TCPC significantly correlate with reduced exercise capacity and increased levels of liver fibrosis/congestion (cT1), while ventricular ejection fraction and cardiac index did not. Liver cT1 values were elevated in all patients indicating universal presence of liver fibrosis/congestion. The Fontan tunnel and LPA were the segments with most adverse energetics indicating potential room for improvement either at time of initial Fontan operation (e.g. implanting expandable conduits) or by intervention stenting/dilatation or possibly conduit replacement. 4D flow CMR is therefore promising as a non-invasive screening tool for identification of patients with adverse TCPC flow efficiency.

## Supplementary Information

**Additional file 1:** **Table S1.** CMR acquisition details. **TableS2**. Correlation analysis between cross-sectional area ofthe TCPC segments and 4D flow CMR energetics. **TableS3.** Correlation analysis between demographic and CMRparameters with exercise capacity and liver cT1 mapping. **Fig. S1.** A flow chart of the study methods and performed analyses.

## Data Availability

The datasets used and/or analyzed during the current study are available from the corresponding author on reasonable request.

## Abbreviations

BSA: Body surface area
CFD: Computational fluid dynamic
CMR: Cardiovascular magnetic resonance
CPET: Cardiopulmonary exercise testing
CSA: Cross-sectional area
cT1: Iron-corrected T1 mapping
CVP: Central venous pressure
ECG: Electrocardiogram
EL: Viscous energy loss rate
KE: Kinetic energy
LPA: Left pulmonary artery
PA: Pulmonary artery
PDFF: Proton density fat-fraction
RPA: Right pulmonary artery
SVC: Superior vena cava
TCPC: Total cavopulmonary connection
VO_2_: Peak oxygen uptake
