# Supplementary material for: 4D flow cardiovascular magnetic resonance derived energetics in the Fontan circulation correlate with exercise capacity and CMR-derived liver fibrosis/congestion
Source: J Cardiovasc Magn Reson. 2022 Mar 28;24:21. doi: 10.1186/s12968-022-00854-4 (PMC8962091; doi:10.1186/s12968-022-00854-4)

| **Additional file 1: Table S1**. CMR acquisition details | | |  |
| --- | --- | --- | --- |
|  | **4D flow CMR** | | |
| No. of slices (orientation) | | 27 (coronal) | |
| Field of view, mm | | 350x271x56 | |
| ECG-gating | | Retrospective | |
| No. of reconstructed cardiac phases | | 24 | |
| No. of signal averages | | 1 | |
| Respiratory compensation | | navigator | |
| Typical navigator efficiency | | 40-60% | |
| Acquired spatial resolution (mm) | | 2.4x2.4x2.4 | |
| Reconstructed spatial resolution (mm) | | 2.1x2.1x2.1 | |
| Acquired temporal resolution, ms (SD) | | 32.0 | |
| Flip angle (°) | | 10 | |
| TE (ms) | | 4.5 | |
| TR (ms) | | 8.0 | |
| VENC (cm/s) | | 80 | |
| Typical scan time (minutes) | | 12-18 | |
| Acceleration methods | | SENSE factor 1.5, RL direction, EPI factor 5, Segmentation factor 1 | |
| SD; standard deviation, ms; milliseconds, mm; millimeter, TE, echo time; TR, repetition time; VENC, velocity encoding; SENSE, sensitivity encoding; AP. anterior-posterior; EPI, echo planar imaging readout; ECG, electrocardiogram | | | |

| **Additional file 1: Table S2**. Correlation analysis between cross-sectional area of the TCPC segments and 4D flow CMR energetics | | | | | | |
| --- | --- | --- | --- | --- | --- | --- |
|  | n | **CSA (mm^2^/m^2^)** | **KE_norm_flow+length_** |  | **EL_norm_flow+length_** |  |
|  |  |  | Correlation coefficient | p | Correlation coefficient | p |
| Fontan tunnel | 35 | 106 (91-134) | -0.80 | **<0.001** | -0.78 | **<0.001** |
| SVC | 31 | 96 (76-109) | 0.23 | 0.21 | -0.02 | 0.93 |
| LPA | 46 | 92 (70-113) | 0.03 | 0.85 | -0.31 | **0.034** |
| RPA | 49 | 88 (81-101) | -0.10 | 0.48 | -0.10 | 0.50 |
| Correlation coefficients represent Pearson or Spearman rank analysis. SVC, superior vena cava; LPA/RPA, left/right pulmonary artery; CSA, cross-sectional area normalized for body surface area; TCPC, total cavopulmonary connection; KE, kinetic energy; EL, viscous energy loss rate | | | | | | |

| **Additional file 1: Table S3.** Correlation analysis between demographic and CMR parameters with exercise capacity and liver cT1 mapping | | | | | | | |
| --- | --- | --- | --- | --- | --- | --- | --- |
|  |  |  |  |  |  |  |  |
|  |  | **cT1** |  | **Peak VO2 (ml/kg/min)** | | **% Predicted peak VO2** | |
| **Demographics** | | Correlation coefficient | p | Correlation coefficient | p | Correlation coefficient | p |
| Age at CMR, years | | 0.254 | **0.048** | -0.42 | **0.003** | -0.29 | **0.039** |
| Time since Fontan, years | | 0.192 | 0.14 | -0.41 | **0.003** | -0.32 | **0.024** |
| LV/biv vs RV |  |  |  | - | 0.76 | - | 0.15 |
| HLHS/non-HLHS | | - | 0.73 | - | 0.96 | - | 0.38 |
| **MR parameters** | |  |  |  |  |  |  |
| Cardiac index, L/min/m^2^ | | 0.079 | 0.55 | 0.25 | 0.08 | -0.07 | 0.61 |
| Ejection fraction, % | | -0.05 | 0.70 | 0.07 | 0.64 | 0.20 | 0.17 |
| *4D flow CMR energetics total TCPC* | | |  |  |  |  |  |
| KEnorm_flow | | 0.50 | **0.006** | -0.61 | **0.003** | -0.44 | **0.04** |
| ELnorm_flow | | 0.39 | **0.04** | -0.54 | **0.009** | -0.46 | **0.033** |
| CMR; cardiovascular magnetic resonance, LV/RV, left/right systemic ventricle, biv; biventricular; HLHS, hypoplastic left heart syndrome; 4D, four dimensional; TCPC, total cavopulmonary connection; KE, kinetic energy; EL, viscous energy loss rate | | | | | | | |

**Additional file 1: Figure S1.**

A flow chart of the study methods and performed analyses.

.


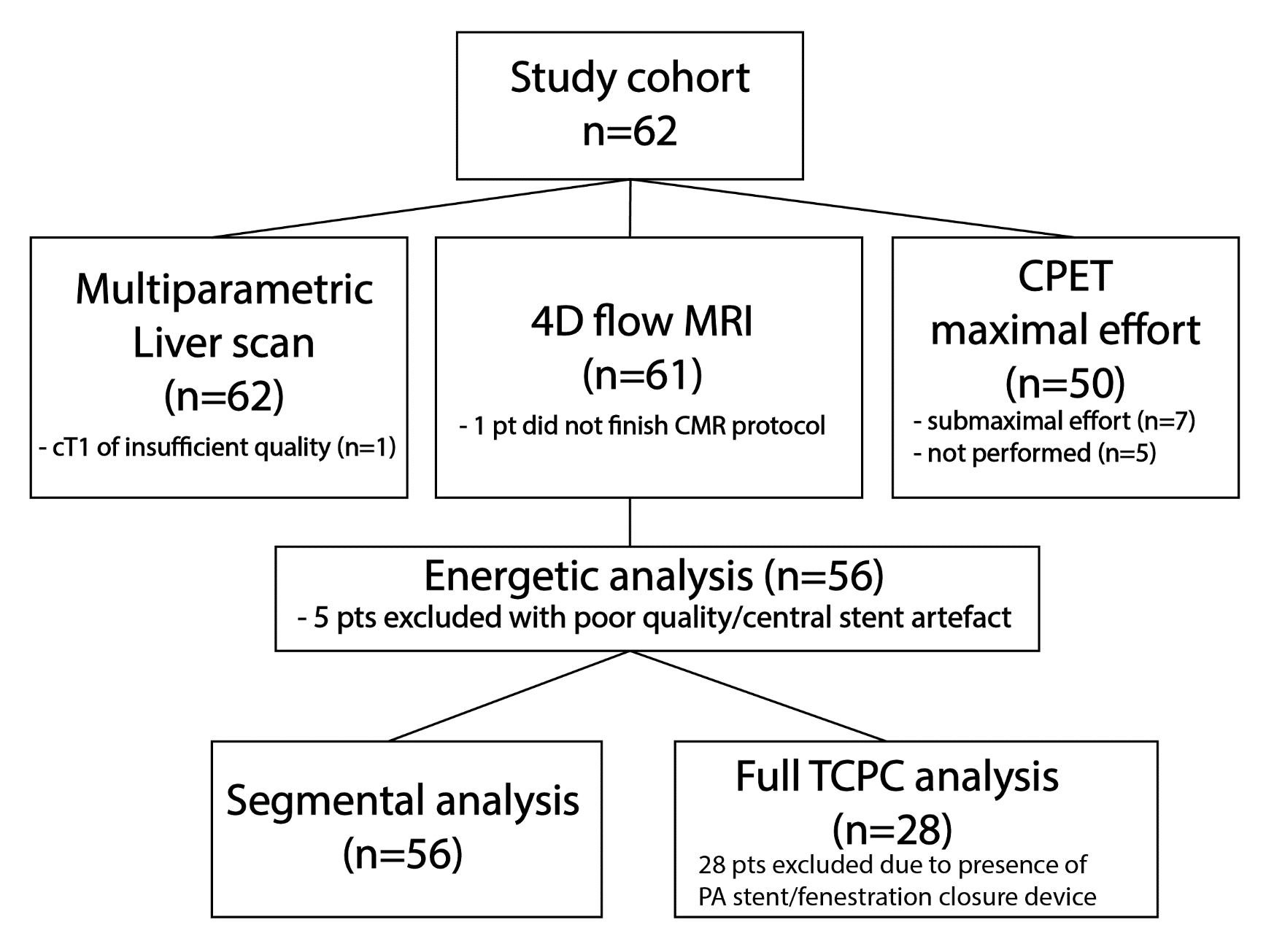

Supplement: Supplementary file 1 — Additional file 1: Table S1. CMR acquisition details. TableS2. Correlation analysis between cross-sectional area ofthe TCPC segments and 4D flow CMR energetics. TableS3. Correlation analysis between demographic and CMRparameters with exercise capacity and liver cT1 mapping. Fig. S1. A flow chart of the study methods and performed analyses. [file 12968_2022_854_MOESM1_ESM.docx]
